# Post-traumatic stress disorder (PTSD) probability among parents who live in Kandahar, Afghanistan and lost at least a child to armed conflict

**DOI:** 10.1038/s41598-023-31228-0

**Published:** 2023-03-10

**Authors:** Mohammad Paiman Rahimi, Mohammad Hashim Wafa, Muhammad Haroon Stanikzai, Bilal Ahmad Rahimi

**Affiliations:** 1 Master of Public Health Program, Faculty of Medicine, Kandahar University, Kandahar, Afghanistan; 2 Neuropsychiatric and Behavioral Science Department, Faculty of Medicine, Kandahar University, Kandahar, Afghanistan; 3 Public Health Department, Faculty of Medicine, Kandahar University, Near Ayno Mena, 10th District, Kandahar, Afghanistan; 4 Pediatrics Department, Faculty of Medicine, Kandahar University, Kandahar, Afghanistan

**Keywords:** Psychology, Neurology, Risk factors

## Abstract

The last 4 decades of conflict in Afghanistan resulted in incalculable deaths, injuries, and millions of displacements. Although there are routine reports on casualties of the warfare, the information on its long-term psycho-social sequelae is somehow discounted. This study aimed to assess post-traumatic stress disorder (PTSD) probability and its associated factors among parents who live in Kandahar, the southern province of Afghanistan, and lost at least one child to armed conflict. We conducted a health-facility-based cross-sectional study involving 474 bereaved parents in Kandahar province from November/2020 to January/2021. The questionnaire was composed of sections on socio-demographic characteristics and mental and medical histories of the parent, features of the traumatic event and the time elapsed since then, age and gender of the lost child, and PCL-5. We performed multivariable logistic analysis to determine factors associated with PTSD probability in such parents. A staggering number of the parents (430; 90.72%) scored > 33 on PCL-5 denoting presence of probable PTSD. We noticed that several attributes of the bereaved parents (rural residence [AOR = 3.71 (95% CI 1.37–9.97)], older age [AOR = 2.41 (95% CI 1.03–5.57)], experiencing more than one traumatic event [AOR = 2.91 (95% CI 1.05–7.94)], pre-existing medical condition [AOR = 3.5 (95% CI 1.55–8.05)], and losing a < 5-years-old child [AOR = 2.38 (95% CI 1.16–4.70)] were significantly associated with PTSD probability. We assert that a very high number of bereaved parents are susceptible to probable PTSD. This finding signifies the eminent necessity of mental health services in such settings and provides implicit insights to relevant humanitarian assistance providers.

## Introduction

The last four decades of conflict in Afghanistan resulted in incalculable deaths, injuries, and millions of displacements^1–3^. Kandahar province was the center for many armed conflicts that resulted in innumerable deaths, injuries, and displacements^1^. Although there are local routine reports on casualties of the warfare, they somehow discount the information on its long-term psychosocial sequelae, such as depression, anxiety, and stress-related disorders. Following a traumatic experience, some people may engage in aggressive behaviors, which in turn may lead to an increased rate of interpersonal conflicts and even social malfunctioning^4–6^. While it is well-known that an intense traumatic exposure, such as encountering combat and/or losing a loved person, leads to the emergence of posttraumatic stress disorder (PTSD) in a large number of cases, the disorder is largely under-reported in bereaved parents^5–7^.

Depending upon PTSD vulnerability factors, such as being female, combat exposure, physical injury, and losing a loved person, the prevalence of PTSD is significantly variable^8–10^. Worldwide, the PTSD prevalence is estimated to be 8% in men and 20% in women^11^. The prevalence of PTSD ranges between 14 and 37% in populations affected by conflict^12–15^. In a study conducted on an Afghan national sample, the prevalence of PTSD was 42.2% and higher among women^16^. This stress-related condition is more prevalent among those who lost a loved one^17,18^.

Studies indicate that parents who were injured physically and lost their children in a traumatic incident are highly prone to mental disorders especially PTSD^17,18^. Such proclivity somehow reflects the bio-psycho-social nature of their infliction. Physical injury (wounds, losing a body part) and its associated phenomena such as pain especially related to warfare are well-known predictors of PTSD^19,20^. Likewise, encountering life threat, experiencing fear or horror in a combat context, and witnessing the injury or death of a loved one in the event, are also well-studied predictors of PTSD^18,21,22^. Finally, minority status, exposure to interpersonal aggression, and living in a combat driven and violence-charged area are considered PTSD risk indicators^19,21^. The individual proclivity to mental disorders, especially PTSD, due to his/her biopsychosocial infliction is, however, not sufficiently signified in pertinent literature on the global and regional scale. Likewise, no recent research has examined the probability of PTSD among parents who lost at least one child to armed conflict in Afghanistan.

This study aimed to address this lacuna by assessing probability of PTSD and its associated factors among parents who live in Kandahar, the southern province of Afghanistan, and lost at least one child to armed conflict. Hence, the study reflects a tiny glimpse of the mental health of bereaved parents who currently live in a very insecure, defenseless, vulnerable and non-immune setting deprived of even basic human needs, such as access to sufficient food, protective housing, environmental safety and appropriate healthcare, to name a few. Additionally, understanding such parents who are vulnerable to PTSD and the external factors that impact their PTSD probability will most probably assist in improving the quality of current interventions delivered in conflict and post-conflict settings.

## Materials and methods

### Study settings and design

We conducted this health-facility-based cross-sectional study in Kandahar province from November/2020 to January/2021. Out of 25 Comprehensive Health Centers (CHCs) operating in Kandahar city and its rural districts, we randomly selected eight CHCs for our data collection. Using the lottery method, we picked four CHCs from the metropolitan area (Nazu Ana CHC, Amer Jan CHC, Fatima Zahra CHC, and Shams-Ul-Haq Kakar CHC) and four CHCs from the rural districts (Maiwand district CHC, Sanzary district CHC, Zharai district CHC, and Wayand district CHC). At the time of inclusion, the aforementioned rural districts were relatively secure to access the CHCs.

### Sample size and sampling procedures

We employed the single population proportion formula [n = Z^2^P(1 − P)/(d)^2^] the assumptions of 95% confidence interval (CI), margin of error = 5%, *P* (prevalence) = 0.5 (the most conservative prevalence estimates)^23^, and 10% non-response rate. The calculated sample size was increased from a minimum of 422 participants to 480 participants in the prevision of missing data. We approached 522 parents who lost a child to armed conflict during their CHC visit. The response rate was 91.1% (476). The final analysis consists of 474 participants with their complete data sets.

### Inclusion and exclusion criteria

Our target population consisted of parents who have lost at least one child to armed conflict and visited one of the participating CHCs. We excluded parents who lost their children due to a factor other than armed conflict, those with compromised consciousness (unable to understand/utter a normal verbal response or not fully awake/alert), and those who were currently somehow under guardianship.

### Study measures

The socio-demographical characteristics of our subjects that were collected included age, gender, marital status, educational background, occupation, and family income.

Trauma-related variables were the number and nature of the traumatic event and the time elapsed since its occurrence. In our study, the index trauma was the child’s death. Other variables of interest comprised a history of any previous mental and or medical conditions and their access to biopsychosocial support. Age and gender were characteristics of the lost child.

In this study, we have employed the term physical threat if, for instance, the event involved the usage of any dangerous weapon, the term emotional threat if the event caused the person to have negative emotions, and the term social threat if the event caused any public demoralization or degrading. We accounted for such biopsychosocial threats in the characteristics of the event section of the questionnaire. Additionally, we employed the term “pre-existing medical condition” to denote exclusively a chronic health problem, such as Diabetes Mellitus (DM), Cardiovascular Diseases (CVD), or Ischemic Heart Diseases (IHD), that the participant might have suffered from prior to the encounter of the index trauma. On the other hand, the study reserved the term “pre-trauma mental health problems” solely for any psychological or emotional problem that the participant has endured prior to the experience of the index trauma. Finally, we employed the term “pre-trauma physical health problem” in a broader sense to imply any physical (medical or surgical) injury, disease, or disability, be it short-lived or long-lived, that the participant has suffered. It neither embraces a medical condition (mentioned earlier) nor a mental health problem.

This study employed the posttraumatic stress disorder checklist for DSM-5 (PCL-5) to measure the presence and severity of PTSD symptoms during the last month and assess the prevalence of probable PTSD. PCL-5 is a 5-point scale from 0 (not at all) to 4 (extremely) that yields a total score from 0 to 80. A score of ≥ 33 on PCL-5 was used to signify susceptibility of bereaved parents to probable PTSD as defined in a prior work^24,25^. PCL-5 is a globally credible instrument with good psychometric properties for assessing PTSD probability in conflict and post-conflict settings^25–27^. The internal consistency (Cronbach’s alpha) value for the Pashtu version in the pre-tested sample was 0.91.

### Data collection

The study questionnaire was initially drafted in English and subsequently translated into the local language (Pashtu) for the convenience of interviewing local participants (Supplementary Files). Prior to the commencement of the study, the questionnaire was pre-tested in a pilot study to check and revise (if required) its verbal consistency and structural reliability.

The data were collected by eight (male and female) experienced nurses/counselors. They attended pre-commencement training sessions on sampling, interviewing, filling out the questionnaires, and addressing potential ethical concerns that may emerge during the study. The interviewer (counselor or medical nurse) screened (based on inclusion criteria) every consecutive patient (non-probability sampling technique) visiting one of the participating CHCs. Then, the recruiters explained the study to eligible patients. Prior to their invitation for participation, the recruiters offered the patients a written synopsis of study objectives and information on the course of the research and that the findings will be publicized. The recruiters declared that participation in this research project would neither change any healthcare the consumer may require nor negates their right to retract from the study at any time. If the patient would like to participate, they could willfully date and sign the consent form. Only then, a trained interviewer collected the baseline data. Female interviewers interviewed all female participants. The recruitment process was supervised by a trained health professional. Daily, we checked the questionnaires for completion.

### Statistical analysis

We employed the statistical package for social sciences (SPSS) version 21 for data analysis^28^. We used descriptive statistics to assess the prevalence of probable PTSD. To determine the associated factors in parents with probable PTSD, we used chi-square statistics. We used a binary logistic regression to determine factors associated with probable PTSD in parents who lost at least one child to armed conflict. We identified and retained into multivariable logistic regression every independent variable with a p-value of less than 0.25. Finally, we performed multivariable logistics regression with the entering method to determine factors associated with probable PTSD in such parents.

### Ethical approval and consent to participate

The Research and Ethics Committee (REC) of Kandahar University approved this study (Letter No: 53; Dated: 28/7/2019). All methods were carried out in accordance with World Medical Association (WMA)’s Declaration of Helsinki. Prior to the commencement of the project, we received approval for data collection from Kandahar Public Health Directorate. Additionally, we provided every potential participant with information concerning the study objectives, benefits/risks of participation, their right to retract from the study at any time they desire and that the data will be published for public awareness and welfare. We obtained informed written consents from every subject.

## Results

### Socio-demographic characteristics of the study participants

We recruited a total of 474 eligible parents of the participating CHCs. The mean age of the study participants was 38 years (± 7.1 years), and nearly 50% (238) were within an age range of 36–50 years. As shown in Table 1, more than half of the respondents were male (269; 56.8%), urban residents (58%), and with no formal education (275; 55.7%). A vast majority of them (392; 82.7%) had a monthly household income of Afghanis < 10,000 (approx. USD 120).

**Table 1:** Socio-demographic characteristics of the study participants (N = 474).

Variables	Frequency (%)
Age (In completed years)	
18–35	53 (11.2)
36–50	238 (50.2)
> 50	183 (38.6)
Gender	
Male	205 (43.2)
Female	269 (56.8)
Residence	
Urban	199 (42)
Rural	275 (58)
Marital status	
Married	408 (86.1)
Widowed	58 (12.1)
Divorced	8 (1.7)
Educational status	
Formal education	80 (16.9)
Religious education	130 (27.4)
No formal education	264 (55.7)
Monthly household income (In Afghanis)	
≤10,000	392 (82.7)
>10,000	82 (17.3)

### Characteristics of the bereaved parents and the index trauma

Of parents interviewed, 410 (86.5%) lost a single child. The children were mostly (324; 68.4%) under five, and in the majority of the cases (385; 81.2%), they were male. In terms of the number and nature of the traumatic event, an almost third (135; 28.5%) of the parents experienced more than one traumatic event, and most of the events were most often included with an emotional threat (224; 47.3%), followed by physical threat (198;41.3%), and social threats (26;5.5%). Prior to the index trauma, 83 (17.5%) respondents were suffering from pre-trauma mental health problems, and 44 (9.3%) of them had pre-trauma medical conditions. Additionally, more than half (283; 59.9%) of the subjects asserted that they had lost other close relatives to armed conflict. Table 2 depicts the characteristics of the respondents and the index trauma.

**Table 2:** Characteristics of the bereaved parents and the index trauma (N = 474).

Variables	Frequency (%)
Number of lost child/children	
One	410 (86.5)
More than one	64 (13.5)
Gender of lost child/children	
Male	385 (81.2)
Female	89 (18.8)
Age of lost child/children	
< 5 years	324 (68.4)
≥ 5 years	150 (31.6)
Time since loss	
≤ 1 year	244 (51.5)
>1 year	230 (48.5)
Reason for referral	
Psychosocial consultation	176 (37.1)
Others	298 (62.9)
Number of events	
One	339 (71.5)
More than one	135 (28.5)
Nature of the experienced threat	
Physical	198 (41.8)
Emotional	224 (47.3)
Social	26 (5.5)
Bio-psycho-social	26 (5.5)
Pre-trauma mental health problems	
Yes	83 (17.5)
No	391 (82.5)
Pre-trauma physical health problems	
Yes	126 (26.6)
No	348 (73.4)
Peri-traumatic use of medications	
Yes	180 (38)
No	294 (62)
Availiability of socio-economic support	
Yes	354 (74.7)
No	120 (25.3)
Pre-existing medical conditions	
Yes	44 (9.3)
No	430 (90.7)
Loss of any other close relative due to war	
Yes	283 (59.9)
No	191 (40.3)

### Probable PTSD prevalence in bereaved parents

**Figure 1:**
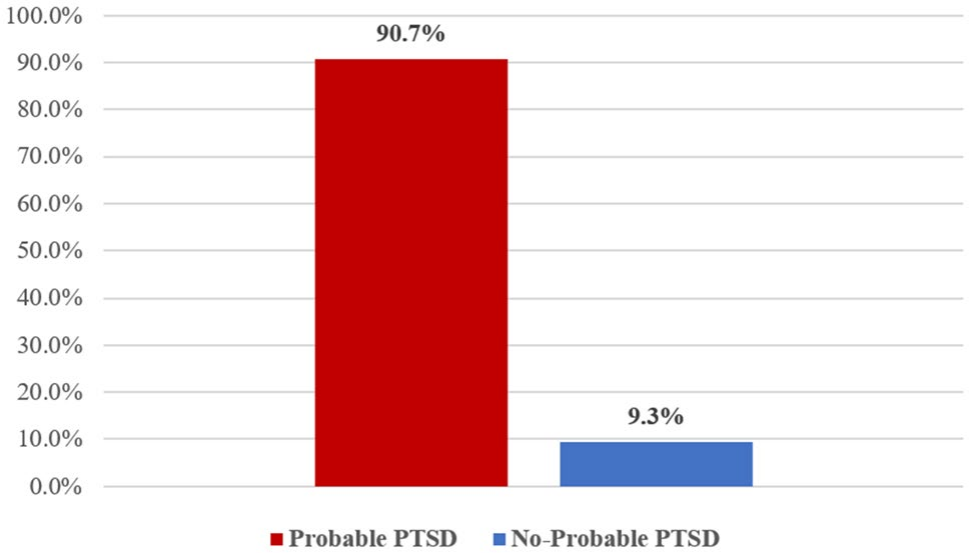
Probable PTSD prevalence in bereaved parents.

A vast majority of our sample of bereaved parents (90.72%; 430) scored ≥ 33 on PCL-5 [95%CI: 87.74%-93.17%] (Fig. 1). The inter- and intra-rater reliability was acceptable (inter-rater reliability > 0.82, intra-rater reliability > 0.91).

### Factors that predicted probable PTSD in our sample of bereaved parents

We conducted the bivariate analysis for all independent variables. Multiple logistic regression analysis was done for those variables with a *P* value of < 0.25 in bivariate analysis. The multiple logistic regression showed that the odds of probable PTSD was higher in parents who lived in rural areas (Adjusted Odds Ratio [AOR] = 3.71, 95% CI 1.37–9.97) than urban residing parents. Besides, parents who were aged > 50 years had 2.41 times higher odds of probable PTSD (AOR 2.41, 95% CI 1.03–5.57) than parents aged ≤ 50. The number of experienced traumatic events was significantly associated with the development of probable PTSD. The odds of developing probable PTSD among parents who have experienced more than one traumatic event were 2.91 times (AOR 2.91, 95% CI 1.05–7.94) more than those who have experienced only one traumatic event. A pre-existing medical condition was also substantially associated with the development of probable PTSD. In contrast to the parent with no pre-existing medical condition, those with a pre-existing medical condition were 3.5 times (AOR 3.5, 95% CI 1.55–8.05) more likely to develop probable PTSD. Furthermore, the age of the lost child was highly associated with the development of probable PTSD. Parents who lost a < 5-years-old child had 2.38 times higher odds (AOR 2.38, 95% CI 1.16–4.70) than parents who lost a ≥ 5-years-old child. Table 3 summarizes the results of the bivariate and multivariable analysis.

**Table 3:** Factors associated with odds of developing probable PTSD in bereaved parents who live in Kandahar Province, 2021; crude and adjusted odds ratio.

Independent variable	Categories	Crude odds ratio (95% CI)	*P*-Value	Adjusted odds ratio (95% CI)	*P*-Value
Respondent’s residence	Rural	6.22 (2.40–16.11)	< 0.001	3.71 (1.37–9.97)	0.01
	Urban	1		1	
Gender of the respondent	Male	1	0.51	–	–
	Female	1.01 (0.89–2.2)		–	
Age of the respondent	< 50 years	1	0.007	1	0.042
	> 50 years	2.99 (1.35–6.60)		2.41 (1.03–5.57)	
Number of the event	1	1	0.014	1	0.039
	> 1	3.28 (1.26–8.52)		2.91 (1.05–7.94)	
Availability of bio-psycho-social problems	Yes	1	0.008	–	–
	No	2.32 (1.21–4.42)		–	
Loss of any other close relative	Yes	1.80 (0.95–3.37)	0.067	–	–
	No	1		–	
Pre-exisiting medical problems	Yes	4.82 (2.26–10.29)	< 0.001	3.5 (1.55–8.05)	0.003
	No	1		1	
Time since the loss	< 1 year	0.60 (0.31–1.14)	0.127	–	–
	> 1 yer	1		–	
Number of the lost child/children	1	1	0.139	–	–
	> 1	1.80 (0.82–3.97)		-	
Age of the lost child/children	< 5 years	3.4 (1.79–6.45)	< 0.001	2.38 (1.16–4.70)	0.002
	> 5 years	1		1	
Gender of the lost child/children	Male	2.58 (1.31–5.07)	0.005	–	–
	Female	1		–	

## Discussion

We aimed to assess PTSD probability in parents residing in Kandahar province and lost at least a child to armed conflict. Our findings suggest the necessity for mental health services in these settings and provide key information that humanitarian assistance providers can use. A staggering number (430; 90.72%) of our study subjects scored ≥ 33. We observed that several attributes of the bereaved parent (i.e., their residential area, age, the number of the traumatic events they encountered, and their pre-existing medical condition) and the age of the lost child were highly associated with PTSD probability.

To the best of our knowledge, the literature on PTSD probability in parents who lost at least a child to armed conflict, and that may provide a context to our findings, is very scarce. However, some studies assessed PTSD symptoms in parents who lost a child to other traumatic events. These studies found higher levels of PTSD symptoms ranging from 12.5 to 63% in their subjects^29–31^.

A limited number of mental health facilities in terms of infrastructure and personnel, the exceptional severity and lengthy duration of warfare, poverty, unemployment, and illiteracy in this geography are some of the factors that may have brought about such a staggering level of PTSD probability. Therefore, the provision of mental health services for this population is of paramount importance.

Our results epitomize the difference in PTSD probability in urban and rural populations. A significantly higher number (194) of the rural population were more susceptible to PTSD than their urban counterparts. This finding has been found in prior studies and may signify the higher level of insecurity, limited access to health facilities, as well as socio-cultural impediments (stigma, and traditional approaches of care) in rural settings^32–34^. Hence, further studies are warranted to explore unique contributors to this higher susceptibility of PTSD in rural populations.

Consistent with relevant literature, our findings indicate that PTSD probability was lower in parents aged ≤ 50 years than those aged beyond 50 years. Studies have found that PTSD probability was higher in bereaved people who lost a loved one in old age^35,36^. This finding may somehow elucidate additional stressors, such as increased bio-psychological health problems, low social support, poor resilience capability, and the loss of independent life that bereaved elders may have to endure. We recommend the provision of biological (medications), monetary (household material, nutrients, money), psychological (counseling), and social (tangible and non-tangible) support to bereaved parents through a patient-oriented sustainable program. Such a program may prove exceptionally productive to control PTSD symptoms, particularly in parents who are beyond 50.

We noticed that bereaved parents who have experienced more than one traumatic event were 2.91 times more susceptible to PTSD. Similar studies on individuals who lost a loved one or experienced other traumatic events have shown an association between the number of traumatic events experienced- either to the same or a different type of event- and PTSD probability^29,30^. An earlier study among the Afghan population also demonstrated an association between mental health status and the number of traumatic events^16^. Despite a 40-year continuous conflict and numerous traumas witnessed by Afghans, the country had not developed robust mental health services either at a facility or national level. Thus, augmenting the availability (quantity) and functionality (quality) of these services is highly recommended.

As indicated in pertinent literature, we found that the presence of a pre-traumatic medical condition was significantly associated with higher PTSD probability^29,37^. Extensive research highlights that the solo presence of a medical condition, such as DM, cancer, is associated with higher PTSD probability^38,39^. Needless to mention that, PTSD symptoms may predispose bereaved parents to enhanced risk of medical problems^40–42^. Hence, screening and early identification of bereaved parents with a pre-existing medical condition are of great importance to circumvent additional medical conditions and their psychosocial sequelae.

We observed that the age of the lost child was significantly associated with PTSD probability. That is, in parents who lost a < 5-years-old child, PTSD probability was 2.38 times higher. In contrast, Yin Q et al. (2018) reported a higher prevalence of PTSD symptoms among parents who lost a child of older age^30^. Hence, this finding is to be further explored by future research.

In this study, the participants of the female gender showed higher rates of PTSD, but it was not statistically significant. However, previous studies reported that women are two to three times as likely as men to meet the criteria for PTSD following such events^16,43,44^. To elaborate on this discrepancy, the following points may prove worthwhile. First, to the best of our knowledge, this is the first study that assessed PTSD probability in Afghanistan, especially in Kandahar province. Therefore, some socio-demographic factors that contribute to the uniqueness of this area, such as Pashtun culture, and discordance of gender roles in western society, may have affected the findings. Second, women tend to communicate more, especially during times of adversity, and are more resilient than men^16,45,46^. Therefore, data from a red zone, such as Kandahar city may bear little concordance with a study conducted in a post-conflict setting or green zone. Third, previous studies have almost always assessed PTSD in post-conflict settings. This may be the first study that evaluated PTSD probability in a current-conflict setting. Fourth, there is a tiny likelihood that the female participants may have adjusted their responses and made the responses appropriate for the group setting to avert potential husband or family retribution, in case they were quite honest in their answers. Fifth, for some understandable reasons, such as security, ease of access, and its well-known address, the CHC, in addition to its health care services, is a typical setting for welfare activities and donation distribution. Therefore, our participants may have unconsciously attuned their responses to attracting or obtaining a potential donation.

## Limitations

We recommend considering our findings in light of the following limitations. Firstly, we did not employ the gold standard diagnostic tool for PTSD, that is, CAPS-5. Besides, we appreciated that clinical interview may have produced a lower percentage, as PCL-5 may be biased and have inflated the positive cases. Therefore, we have used a more conservative label (probable PTSD). Secondly, we did not measure “prolonged grief” and “depression” in our subjects. This in turn may have overlap and somehow altered our results. Thirdly, our subjects represent physically and/or emotionally distressed parents who presented to the CHC for care. Therefore, such high rates (90.72%) of probable PTSD in clinical population may not represent the general population. Fourthly, the research articles that could provide a benchmarking context to our findings were very scarce, particularly in rural populations. Fifthly, this was the first attempt to use the Pashtu version of PCL-5, while its psychometric properties are yet to be validated. Sixthly, the nearly four decades-long persistent warfare in Afghanistan and its inevitable multitude of consequences contribute immensely to the unique attributes of our study setting and subjects and the reduction of our results generalizability. It somehow elucidates the staggering number (430; 90.72%) of our study participants susceptible to PTSD. Seventhly, we have not accounted for subjects with subthreshold symptoms of PTSD who scored < 33. Finally, our results reflect a deficiency of assessing diverse levels (low, medium, or high) of PTSD probability.

## Conclusion

We concluded that bereaved parents who lived in Kandahar province and lost a child to the armed conflict were highly (430; 90.72%) susceptible to probable PTSD. Our study, conducted in a combat-ridden setting, has come across some respondent-related factors. These factors were significantly associated with higher PTSD probability. They include parents’ attributes such as residential area (rural vs. urban), age, number of traumatic events they have experienced, and their pre-traumatic medical condition, as well as the age of the lost child.

## Supplementary Information

Supplementary Information.

## Data Availability

The primary data used to support the findings of this study are available with the corresponding author upon request.

## References

[1] Burki T (2016). Conflict in Afghanistan takes an increasing toll on civilians. Lancet.

[2] Thompson DC, Crooks RJ, Clasper JC, Lupu A, Stapley SA, Cloke DJ (2017). The pattern of paediatric blast injury in Afghanistan. BMJ Mil. Health.

[3] Cameron CM, O’Leary PJ, Lakhani A, Osborne JM, de Souza L, Hope K (2018). Violence against children in Afghanistan: Community perspectives. J. Interpers. Violence.

[4] Thomas MM, Harpaz-Rotem I, Tsai J, Southwick SM, Pietrzak RH (2017). Mental and physical health conditions in US combat veterans. Prim. Care Companion CNS Disorders.

[5] Bogic M, Njoku A, Priebe S (2015). Long-term mental health of war-refugees: A systematic literature review. BMC Int. Health Hum. Rights.

[6] Tay AK, Riley A, Islam R, Welton-Mitchell C, Duchesne B, Waters V (2019). The culture, mental health and psychosocial wellbeing of Rohingya refugees: A systematic review. Epidemiol. Psychiatr. Sci..

[7] Herringa RJ (2017). Trauma, PTSD, and the developing brain. Curr. Psychiatry Rep..

[8] Auxéméry Y (2018). Post-traumatic psychiatric disorders: PTSD is not the only diagnosis. La Presse Médicale.

[9] Brewin CR, Andrews B, Valentine JD (2000). Meta-analysis of risk factors for posttraumatic stress disorder in trauma-exposed adults. J. Consult. Clin. Psychol..

[10] Ehlers A (2012). Post-traumatic stress disorder. New Oxford Textbook of Psychiatry.

[11] Bobo WV, Warner CH, Warner CM (2007). The management of post traumatic stress disorder (PTSD) in the primary care setting. South. Med. J..

[12] Cardozo BL, Vergara A, Agani F, Gotway CA (2000). Mental health, social functioning, and attitudes of Kosovar Albanians following the war in Kosovo. JAMA.

[13] Mollica RF, Sarajlić N, Chernoff M, Lavelle J, Vuković IS, Massagli MP (2001). Longitudinal study of psychiatric symptoms, disability, mortality, and emigration among Bosnian refugees. JAMA.

[14] De Jong JT, Komproe IH, Van Ommeren M, El Masri M, Araya M, Khaled N, van De Put W, Somasundaram D (2001). Lifetime events and posttraumatic stress disorder in 4 postconflict settings. JAMA.

[15] Murthy RS, Lakshminarayana R (2006). Mental health consequences of war: A brief review of research findings. World Psychiatry.

[16] Kovess-Masfety V, Keyes K, Karam E, Sabawoon A, Sarwari BA (2021). A national survey on depressive and anxiety disorders in Afghanistan: A highly traumatized population. BMC Psychiatry.

[17] Chan CLW, Wang C-W, Ho AHY, Qu Z-Y, Wang X-Y, Ran M-S (2012). Symptoms of posttraumatic stress disorder and depression among bereaved and non-bereaved survivors following the 2008 Sichuan earthquake. J. Anxiety Disorders.

[18] Wang Q, Zhang S, Wang Y, Jing Z, Zhou Y, Qi K (2021). Prevalence and risk factors of posttraumatic stress disorder among Chinese shidu parents: A systemic review and meta-analysis. J. Affecti. Disorders.

[19] Heath NM, Chesney SA, Gerhart JI, Goldsmith RE, Luborsky JL, Stevens NR (2013). Interpersonal violence, PTSD, and inflammation: Potential psychogenic pathways to higher C-reactive protein levels. Cytokine.

[20] Tsujiuchi T, Yamaguchi M, Masuda K, Tsuchida M, Inomata T, Kumano H, Kikuchi Y, Augusterfer EF, Mollica RF (2016). High prevalence of post-traumatic stress symptoms in relation to social factors in affected population one year after the fukushima nuclear disaster. PLoS ONE.

[21] Walsh K, Nugent NR, Kotte A, Amstadter AB, Wang S, Guille C (2013). Cortisol at the emergency room rape visit as a predictor of PTSD and depression symptoms over time. Psychoneuroendocrinology.

[22] Powers MB, Warren AM, Rosenfield D, Roden-Foreman K, Bennett M, Reynolds MC (2014). Predictors of PTSD symptoms in adults admitted to a Level I trauma center: A prospective analysis. J. Anxiety Disord..

[23] Bujang MA (2021). A step-by-step process on sample size determination for medical research. Malays. J. Med. Sci..

[24] Weathers, F. W., Litz, B., Herman, D., Juska, J., Keane, T. PTSD Checklist—Civilian Version. PsycTESTS Dataset [Internet]. American Psychological Association (APA); DOI: 10.1037/t02622-000 (1993).

[25] Blevins CA, Weathers FW, Davis MT, Witte TK, Domino JL (2015). The posttraumatic stress disorder checklist for DSM-5(PCL-5): Development and initial psychometric evaluation. J. Trauma. Stress.

[26] Van de Vijver F, Hambleton RK (1996). Translating tests. Eur Psychol..

[27] Sveen J, Bondjers K, Willebrand M (2016). Psychometric properties of the PTSD Checklist for DSM-5: A pilot study. Eur. J. Psychotraumatol..

[28] International Business Machines Corporation, IBM SPSS Statistics for Windows, Version 21.0, IBM Corporation, Armonk, NY, USA. (2012).

[29] Yin Q, Zhang H, Shang Z, Wu L, Sun Z, Zhang F (2020). Risk factors for PTSD of Shidu parents who lost the only child in a rapid aging process: A cross-sectional study. BMC Psychiatry.

[30] Yin Q, Shang Z, Zhou N, Wu L, Liu G, Yu X (2018). An investigation of physical and mental health consequences among Chinese parents who lost their only child. BMC Psychiatry.

[31] Dyregrov K, Dyregrov A, Kristensen P (2014). Traumatic bereavement and terror: The psychosocial impact on parents and siblings 1.5 years after the July 2011 Terror Killings in Norway. J. Loss Trauma.

[32] Qeshta HA, Al Hawajri AM, Thabet AM (2019). The relationship between war trauma, PTSD, anxiety and depression among adolescents in the Gaza strip. Health Sci. J..

[33] Duke MR, Moore RS, Ames GM (2011). PTSD treatment-seeking among rural Latino combat veterans: A review of the literature. J. Rural Social Sci..

[34] Schaffer ME, Crabtree M, Bennett EA, Mcnally M, Okel A (2011). Identifying barriers to treatment for PTSD among reserve component combat veterans in rural. J. Rural Commun. Psychol..

[35] Hoge CW, Castro CA, Messer SC, McGurk D, Cotting DI, Koffman RL (2004). Combat duty in Iraq and Afghanistan, mental health problems, and barriers to care. N. Engl. J. Med..

[36] O’Connor M (2010). A longitudinal study of PTSD in the elderly bereaved: Prevalence and predictors. Aging Mental Health.

[37] O’Connor M (2010). PTSD in older bereaved people. Aging Mental Health.

[38] Cook, J. M. Post-traumatic stress disorder in older adults. PsycEXTRA Dataset [Internet]. American Psychological Association (APA). 10.1037/e400352008-001 (2001).

[39] Steil RI, Hinckers A, Bohus M (2007). Comorbidity of personality disorders and posttraumatic stress disorder. Eur. Psychiatry.

[40] Greene T, Neria Y, Gross R (2016). Prevalence, detection and correlates of PTSD in the primary care setting: A systematic review. J. Clin. Psychol. Med. Settings.

[41] Visser E, Gosens T, Den Oudsten BL, De Vries J (2017). The course, prediction, and treatment of acute and posttraumatic stress in trauma patients. J. Trauma Acute Care Surg..

[42] Cheng Y, Wang F, Wen J, Shi Y (2014). Risk factors of post-traumatic stress disorder (PTSD) after Wenchuan earthquake: A case control study. PLoS ONE.

[43] Bonanno GA, Wortman CB, Lehman DR, Tweed RG, Haring M, Sonnega J, Carr D, Nesse RM (2002). Resilience to loss and chronic grief: A prospective study from preloss to 18-months postloss. J. Personal. Soc. Psychol..

[44] Farhood L, Fares S, Hamady C (2018). PTSD and gender: Could gender differences in war trauma types, symptom clusters and risk factors predict gender differences in PTSD prevalence?. Arch. Women’s Mental Health.

[45] Olff M (2017). Sex and gender differences in post-traumatic stress disorder: An update. Eur. J. Psychotraumatol..

[46] Martínez-Moreno A, Ibáñez-Pérez RJ, Cavas-García F, Cano-Noguera F (2020). Older adults’ gender, age and physical activity effects on anxiety, optimism, resilience and engagement. Int. J. Environ. Res. Public Health.

