# Supplementary material for: Post-traumatic stress disorder (PTSD) probability among parents who live in Kandahar, Afghanistan and lost at least a child to armed conflict
Source: Sci Rep. 2023 Mar 10;13:3994. doi: 10.1038/s41598-023-31228-0 (PMC10006089; doi:10.1038/s41598-023-31228-0)
Supplement: Supplementary file 1 — Supplementary Information. [file 41598_2023_31228_MOESM1_ESM.pdf]

Post-Traumatic Stress Disorder (PTSD) probability among parents who live in Kandahar, Afghanistan and lost at least a child to armed conflict

Authors

[1] Mohammad Paiman Rahimi (paiman.kbdc@gmail.com)

[1,2] Mohammad Hashim Wafa (wafahashim@gmail.com)

[1,3*] Muhammad Haroon Stanikzai (haroonstanikzai1@gmail.com)

[1,4] Bilal Ahmad Rahimi (bilal.rahimi.bilal@gmail.com)

Affiliations

[1] Master of Public Health Program, Faculty of Medicine, Kandahar University, Kandahar, Afghanistan

[2] Neuropsychiatric and Behavioral Science Department, Faculty of Medicine, Kandahar University, Kandahar, Afghanistan

[3] Public Health Department, Faculty of Medicine, Kandahar University, Kandahar, Afghanistan

[4] Pediatrics Department, Faculty of Medicine, Kandahar University, Kandahar, Afghanistan

Dr. Muhammad Haroon Stanikzai, MD, MPH

(Corresponding contributor)

Address: Medical Faculty, Near Ayno Mena, 10th District, Kandahar, Afghanistan

E-mail – haroonstanikzai1@gmail.com

Questionnaire No: …………………………….

**Patient ID #**			
**Date of interview**			
**Name of CHC**			
**Age and age range**	1. (18-35) years ☐	2. (36-50) years ☐	3. Above 50 ☐
**Gender**	1. Male ☐		2. Female ☐
**Residency**	1. Urban ☐	2. Rural ☐	3. Other province ☐
**Current Marital status**	1. Married ☐	2. Widowed ☐	2. Divorced ☐

A. Demographic profile:

B. Socioeconomic status:

**Education Level**	1. Primary ☐	3. High school ☐	5. Religious studies ☐
	2. Secondary ☐	4. Bachelor ☐	6. None ☐
**Economic status**	1. Low (<10000af per month) ☐	2. Middle (10000-20000af per month) ☐	2. High (>20000af per month) ☐
**Employment status**	1. Self-employed ☐	2. Jobless ☐	3. Government job ☐

C. PTSD Checklist for DSM-5 (PCL-5) with Criterion A

Instructions: This questionnaire asks about problems you may have had after a very stressful experience involving actual or threatened death, serious injury, or sexual violence. It could be something that happened to you directly, something you witnessed, or something you learned happened to a close family member or close friend. Some examples are a serious accident; fire; disaster such as a hurricane, tornado, or earthquake; physical or sexual attack or abuse; war; homicide; or suicide.

First, please answer a few questions about your worst event, which for this questionnaire means the event that currently bothers you the most. This could be one of the examples above or some other very stressful experience. Also, it could be a single event (for example, a car crash) or multiple similar events (for example, multiple stressful events in a war-zone or repeated sexual abuse).

Information of bereaved parent(s):

1	Reason of referral?	Psycho-social ☐	Medical problem ☐	Other ☐	
2	Number of event(s)?	Once ☐	Or more than once ☐		
3	Type of the event(s)	1. Physical ☐	2. Emotional ☐	3. Social ☐	4.other (financial, ☐

	to parent(s)?	threat	threat	threat	possession,….)
4	Pre-trauma mental Problem?			1. Yes ☐	2. No ☐
5	Pre-trauma physical illness?			1. Yes ☐	2. No ☐
6	Peritraumatic use of medication?			1. Yes ☐	2. No ☐
7	Available biopsychosocial and financial support?			1. Yes ☐	2. No ☐
8	Peritraumatic use of psychotropic medication?			1. Yes ☐	2. No ☐
9	Loss of any other close relative due to war?			1. Yes ☐	2. No ☐
10	Pre-existing medical conditions such as DM, CVD, CHD?				
11	How long ago did it happened (time since loss)?	Within 3 months ☐		Within 1yr ☐	> 1yr ☐

Information of lost child/children:

1	# of lost child/children due to armed conflict?	One ☐	More than one ☐
2	Age of lost child/children?	<5 ☐	>5 ☐
3	Gender of lost child?	Male ☐	Female ☐

Second, below table is a list of problems that people sometimes have in response to a very stressful experience. Keeping your worst event in mind, please read each problem carefully and then circle one of the numbers to the right to indicate how much you have been bothered by that problem in the past month/ months.

**S/N**	**In the past month, how much were you bothered by:**	**Not at all**	**A little bit**	**Moderately**	**Quite a bit**	**Extremely**
		0	1	2	3	4
1	Repeated, disturbing, and unwanted memories of the stressful experience?					
2	Repeated, disturbing dreams of the stressful experience?					
3	Suddenly feeling or acting as if the stressful experience were actually happening again (as if you were actually back there reliving it)?					
4	Feeling very upset when something reminded you of the stressful experience?					
5	Having strong physical reactions when something reminded you of the stressful experience (for example, heart pounding, trouble breathing, sweating)?					
6	Avoiding memories, thoughts, or feelings related to the stressful experience?					
7	Avoiding external reminders of the stressful experience (for example, people, places, conversations, activities, objects, or situations)?					
8	Trouble remembering important parts of the stressful experience?					

9	Having strong negative beliefs about yourself, other people, or the world (for example, having thoughts such as: I am bad, there is something seriously wrong with me, no one can be trusted, the world is completely dangerous)?					
10	Blaming yourself or someone else for the stressful experience or what happened after it?					
11	Having strong negative feelings such as fear, horror, anger, guilt, or shame?					
12	Loss of interest in activities that you used to enjoy?					
13	Feeling distant or cut off from other people?					
14	Trouble experiencing positive feelings (for example, being unable to feel happiness or have loving feelings for people close to you)?					
15	Irritable behavior, angry outbursts, or acting aggressively?					
16	Taking too many risks or doing things that could cause you harm?					
17	Being "super alert" or watchful or on guard?					
18	Feeling jumpy or easily startled?					
19	Having difficulty concentrating?					
20	Trouble falling or staying asleep?					
